# Optical nonlinearity goes ultrafast in 2D semiconductor-based nanocavities

**DOI:** 10.1038/s41377-022-00827-3

**Published:** 2022-05-07

**Authors:** Armando Genco, Giulio Cerullo

**Affiliations:** Dipartimento di Fisica, Politecnico di Milano, Piazza Leonardo da Vinci 32, 20133 Milano, Italy

**Keywords:** Near-infrared spectroscopy, Ultrafast photonics

## Abstract

Hybrid systems of silver nanodisks strongly coupled to monolayer tungsten-disulfide (WS_2_) show giant room-temperature nonlinearity due to their deeply sub-wavelength localized nature, resulting in ultrafast modifications of nonlinear absorption in a solid-state system.

Decades of scientific progress in the fields of micro and nano-photonics allowed to harness strong interactions between light and matter in solid-state systems, leading to fascinating phenomena and ground-breaking technological applications. Light-matter interactions in semiconductors can be precisely controlled by tuning the coupling between excitons, bosonic quasi-particles made of bound electron-hole pairs, and photons confined in an optical resonator. At high coupling strengths, resonant photons coherently exchange energy with excitons at a rate (Rabi frequency, Ω_R_) higher than that of the dephasing processes (i.e., the rate at which photons escape from the cavity or excitons dephase), entering the so-called strong coupling (SC) regime. From this process, new hybrid quantum states are formed, called polaritons. Reaching the SC regime opened new avenues for observing highly nonlinear optical phenomena in the solid state, such as Bose-Einstein condensation^1,2^, polariton lasing^3,4^ and optical parametric amplification^5^. These discoveries have been recently exploited for the creation of all-optical logic gates^6^ and polariton-based neural networks^7^.

Among the different types of semiconductors systems, monolayers of transition metal dichalcogenides (TMDs) stand out as a highly promising platform for exploring the nonlinear effects produced in the SC regime. Charge carriers in such materials experience an out-of-plane quantum confinement generated by their intrinsic 2D structure which, together with the reduced Coulomb screening, leads to excitons with huge binding energies (several hundreds of meV) and prominent oscillator strengths, persisting up to room temperature^8,9^. Moreover, the broken spatial inversion symmetry of the 2D lattice, together with a large spin-orbit coupling, provides an uncommon pseudo-spin degree of freedom for excitons in the K and K′ valleys of the Brillouin zone. Valley excitons thus become selectively addressable by exciting them with oppositely circularly polarized light^10^, whose valley coherence can be even enhanced by the strong coupling with long-living cavity photons^11^. Excitons in TMDs uniquely combine high temperature stability with large nonlinearities, which makes them an ideal solution for room-temperature polaritonics.

In order to reach the SC regime, TMD monolayers can be coupled to different optical resonators. Seminal studies of TMD polaritons exploited atomically thin flakes embedded in vertical microcavities (Fig. 1a). Such devices consist of two highly reflective planar or concave mirrors facing each other and distanced hundreds of nanometers, confining photons at discrete energy levels, the “cavity modes”, whose frequencies are determined by the distance between the mirrors. The cavity mirrors can be made of metallic films or of Distributed Bragg Reflector (DBR), a sequence of pairs of dielectric layers with high/low refractive index. In such devices, SC was demonstrated for neutral excitons^12,13^, trions^14,15^ and excited Rydberg excitons^16^, using monolayers either mechanically exfoliated or grown by chemical vapor deposition on a large scale^17^. TMD polaritons have been also observed in planar architectures, towards on-chip optical circuits, exploiting the sub-diffraction spatial light confinement of plasmonic nanoresonators (Fig. 1b)^18,19^, or involving propagating modes, as for waveguides^20^, Bloch Surface Waves (BSW)^21^, surface lattice resonances^22,23^, photonic crystal slabs (1D gratings)^24^ (Fig. 1c) and Bound states In the Continuum (BIC)^25^.

**Fig. 1:**
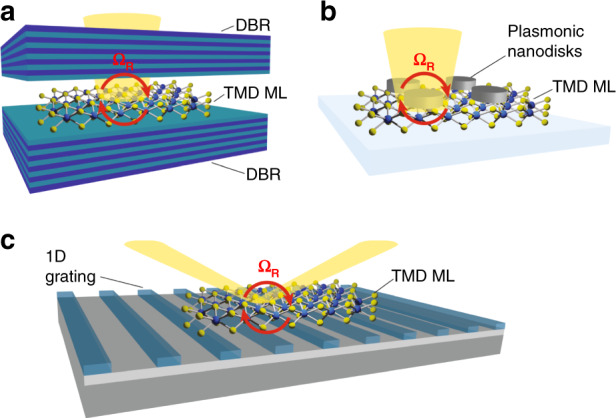
Optical Resonators strongly coupled to TMDs. **a** Schematic of a vertical cavity made of DBRs, embedding a TMD monolayer (ML) in strong coupling regime. **b** Scheme of a plexciton system with a TMD monolayer strongly coupled to silver plasmonic nanodisks. **c** Sketch of a photonic crystal slab made of a 1D grating coupled to a TMD monolayer

Studies of polariton nonlinearities are of key importance for the understanding of the nonlinear optical processes which form the basis of coherent emission, for ultrafast optical switches and for optical parametric scattering, the latter being observed very recently in a TMD vertical cavity^26^. However, investigations of such effects on the ultrafast timescale and in deep-subwavelength optical structures have been very limited so far.

Tang et al.^18^ explore the ultrafast dynamics of the nonlinear optical response for WS_2_ monolayers strongly coupled to plasmonic silver nanodisks, unveiling a giant nonlinearity and studying the underlying physical processes. The non-equilibrium dynamics of the samples were analysed by using femtosecond pump-probe spectroscopy, revealing different exciton-plasmon nonlinear processes over distinct timescales. The main mechanisms which govern the optical nonlinearity in TMD-based microcavity polariton devices^27^ are identified as (i) the repulsive Coulombic interaction between excitons, which usually reflects in a blueshift of the resonance energy, and (ii) phase space filling (or Pauli blocking), inducing excitonic saturation and reducing the exciton-photon coupling strength. In addition, excitation-induced dephasing in TMDs has been recently found to play a non-negligible role at high fluences^28^, typically giving rise to a broadening of excitonic linewidths. The prominent role of the latter mechanism was demonstrated in this work, studying the nonlinear behaviour of plasmon-exciton polaritons (also known as plexcitons) as a function of the excitation fluence. Very interestingly, the authors show a direct application of the strong nonlinear interactions that they found, exploiting the flexibility of the system to easily modify the light-matter coupling strength. This resulted in the observation of direct and reverse absorption saturation at ultrafast timescales, which showcase the potential of TMD polaritons for future uses in sub-wavelength nonlinear optical devices.

There is ample room for further ultrafast studies of the nonlinear optical response of TMD polaritons, from the demonstration of ultrafast all-optical switches working in a low excitation density regime to the holy grail of time-domain observation of Rabi oscillations at the frequency Ω_R_^29^. Recently, it has been shown that greatly enhanced polariton nonlinearities can be achieved using moiré-lattice excitons in twisted TMD hetero-bilayers^30^ and hybridized dipolar excitons in homo-bilayers^31^; it will be interesting to study the out-of-equilibrium optical response for those systems. An additional enticing perspective is to extend the investigation of ultrafast exciton-polariton nonlinearities to other systems featuring strongly bound excitons, such as three- and two-dimensional perovskites^32^.
